# Efficacy of Combined *in-vivo* Electroporation-Mediated Gene Transfer of VEGF, HGF, and IL-10 on Skin Flap Survival, Monitored by Label-Free Optical Imaging: A Feasibility Study

**DOI:** 10.3389/fsurg.2021.639661

**Published:** 2021-03-23

**Authors:** S. Morteza Seyed Jafari, Fabian Blank, Hallie E. Ramser, Alan E. Woessner, Maziar Shafighi, Thomas Geiser, Kyle P. Quinn, Robert E. Hunger, Amiq Gazdhar

**Affiliations:** ^1^Department of Dermatology, Inselspital, Bern University Hospital, Bern, Switzerland; ^2^Department for BioMedical Research (DBMR), University of Bern, Bern, Switzerland; ^3^Department of Pulmonary Medicine, Inselspital, Bern University Hospital, Bern, Switzerland; ^4^Department of Biomedical Engineering, University of Arkansas, Fayetteville, AR, United States; ^5^Hirslanden Klinik, Bern, Switzerland

**Keywords:** cell metabolism, flap survival, gene delivery, HGF, IL-10, *in-vivo* electroporation, label free fluorescence lifetime imaging, VEGF

## Abstract

Preventing surgical flaps necrosis remains challenging. Laser Doppler imaging and ultrasound can monitor blood flow in flap regions, but they do not directly measure the cellular response to ischemia. The study aimed to investigate the efficacy of synergistic *in-vivo* electroporation-mediated gene transfer of interleukin 10 (IL-10) with either hepatocyte growth factor (HGF) or vascular endothelial growth factor (VEGF) on the survival of a modified McFarlane flap, and to evaluate the effect of the treatment on cell metabolism, using label-free fluorescence lifetime imaging. Fifteen male Wistar rats (290–320 g) were randomly divided in three groups: group-A (control group) underwent surgery and received no gene transfer. Group-B received electroporation mediated hIL-10 gene delivery 24 h before and VEGF gene delivery 24 h after surgery. Group-C received electroporation mediated hIL-10 gene delivery 24 h before and hHGF gene delivery 24 h after surgery. The animals were assessed clinically and histologically. In addition, label-free fluorescence lifetime imaging was performed on the flap. Synergistic electroporation mediated gene delivery significantly decreased flap necrosis (*P* = 0.0079) and increased mean vessel density (*P* = 0.0079) in treatment groups B and C compared to control group-A. NADH fluorescence lifetime analysis indicated an increase in oxidative phosphorylation in the epidermis of the group-B (*P* = 0.039) relative to controls. These findings suggested synergistic *in-vivo* electroporation-mediated gene transfer as a promising therapeutic approach to enhance viability and vascularity of skin flap. Furthermore, the study showed that combinational gene therapy promoted an increase in tissue perfusion and a relative increase in oxidative metabolism within the epithelium.

## Introduction

Post-surgical flaps necrosis remains a challenge for the surgeons (1). The promising results of recent studies demonstrate the importance of growth factors in aiding tissue repair and regeneration (1, 2). Application of novel techniques like non-viral gene therapy could further increase the effectiveness by providing sustained therapeutic level of growth factors locally at the skin flap (1, 3). Among various gene delivery methods, nonviral electroporation mediated technique has been shown to be safe and effective for prolonged transgene expression (4–6). *In-vivo* electroporation is based on the principle of enhanced plasma membrane permeability of the tissue upon application of short electric pulses of high voltage, resulting in enhanced DNA uptake (7). Previous preclinical studies by others and us have successfully shown promising results of growth factors like Hepatocyte growth factor (HGF), Vascular endothelial growth factor (VEGF), and Interleukin 10 (IL-10) in attenuating graft necrosis (4–6, 8, 9). Prolonged amelioration of acute rejection in rat lung transplant model using IL-10 and HGF has been demonstrated (10). However, their combined effect on skin graft survival, necrosis, and vascularity has not been reported. Also, Laser Doppler imaging, ultrasound, and fluorescence angiography have been used to monitor blood flow in flap regions (6, 11, 12), but they could not directly measure cellular response to ischemia. Multiphoton microscopy is well-suited for non-invasively monitoring skin metabolism through the intrinsic fluorescence of NADH. Through fluorescence lifetime imaging (FLIM), the protein binding status of NADH can be assessed by measuring the time between excitation and emission (13–15).

In the current study, we investigated the synergistic effects of *in vivo* electroporation-mediated gene transfer of IL-10 with either VEGF or HGF on reduction of skin flap necrosis. Furthermore, the effect of treatment on cell metabolism, using label-free fluorescence lifetime imaging was evaluated.

## Materials and Methods

### Plasmids

Plasmid encoding human VEGF165 isoform (pVEGF) was kindly provided by Prof. Richard Heller (Old Dominion University, Norfolk, VA 23508, USA). The plasmid dissolved in 0.9% saline was commercially prepared (Aldevron, Fargo, ND, USA) to ensure quality. The human pVEGF165 with a pVAX1 backbone and a hEF1-HTLV promoter as described (4). Furthermore, the full-length sequence of human HGF (hHGF) was kindly provided by Prof. Toshikazu Nakamura, Osaka, Japan. The plasmid was constructed by inserting hHGF cDNA (2.1 kb) into the backbone of pCik, driven by the human cytomegalovirus early promoter enhancer (pCikhHGF) between the NotI and NheI sites as described (16). In addition, plasmid pCik IL-10 was constructed by inserting hIL-10 c DNA (537 bp) into a unique backbone of pCIk driven by human CMV early promoter enhancer at Not 1 and Nhe 1 site (17). The plasmids were purified and produced in the quantity required at (Plasmid Factory Gmbh & Co, Bielefeld Germany). All plasmids were dissolved in endotoxin free water for application.

### Animals

Fifteen male Wistar rats (290–320 g) were used in this study protocol and were obtained from Charles River Laboratories (Sulzfeld, Germany). The rats were kept in individual cages in a temperature-controlled and light-controlled animal facility and standard food and water were provided *ad libitum*.

### Study Groups

The animals were randomized into three groups. As the electroporation alone (without gene transfer) did not provide any effect on the outcome in the previous studies (5, 10, 18), the control group A (*n* = 5) underwent the surgery and received no treatment as described previously (4). In the current study we did not include the groups with single gene transfer since the data related to single gene transfer has already been published before hHGF, hIL 10, and hVEGF (6, 9, 19). Group B received electroporation mediated IL-10 gene delivery 24 h before and hVEGF gene delivery 24 h after the surgery (*n* = 5) (IL-10/VEGF group). Group C received hIL-10 gene delivery 24 h before and hHGF gene delivery 24 h after the surgery (*n* = 5) (IL-10/HGF group). Animals were sacrificed seven days later as described below.

### Electroporation Mediated Gene Transfer to the Flap

For gene delivery, the plasmid was suspended in endotoxin- free water at concentration of 1 μg/μl. Total of 100 μg of plasmid (100 μL solution) was injected intradermally (one midline longitudinal injection 1.5 cm away from the edge of the flap) using a 25-gauge needle, at this place usually the necrosis and rejection occurs predominantly (4, 6). After injection of plasmid *in-vivo* electroporation-mediated gene transfer was performed at the site of plasmid injection (midline: 1.5 cm from the edge of the flap) with a series of eight pulses of 200 V/cm, for 10 ms, using a stainless tweezertrode Electrode, 10 mm diameter. NEPA 21electroporator, Sonidel Japan). The distance between the electrodes was 5 mm; the flap was held in between the tweezer electrode and gentle constant pressure was applied during the electroporation procedure to hold the flap (6, 9).

### Surgical Procedure

For the surgical model, a modified McFarlane flap, skin flap on the dorsum of the rat, was used as the surgical model in the current study as described previously (6, 20).

### Evaluations

#### Skin Flap Survival Assessment (Planimetry)

Flap necrosis was quantified by measuring the percentage flap survival, 7 days after surgery. Regions of pink and pliable soft skin with evidence of new hair growth were considered healthy, while regions of thickened, contracted, hard, and dark-colored tissue without new hair growth were considered necrotic for planimetric analyses as described before (21). Image J Software (NIH, Bethesda, MD, USA) was used to calculate the percentage of flap necrosis after analyzing the digital images of each skin flap as previously described (4, 22):

$$\begin{array}{l} \text{Percentage of necrosis }\left(\text{\%}\right)=\;\frac{\text{Area of necrosis }}{\text{Area of the whole flap}\times 100} \end{array}$$

#### Tissue Collection Histology and Immunohistochemistry

At day 7, the animals were sacrificed by intraperitoneal injection of 50 mg/kg of pentobarbital. After resection of the skin flap with the underlying tissue, transverse segments (5 × 5 mm) were taken 12.5–17.5 mm (cranial part) and 72.5–77.5 mm (caudal part) from the cranial margin of the flap. Furthermore, a third segment was taken from the necrosis-survival margin (intermediate part). Additionally, in order to assess possible side effects of gene transfer, kidney and liver samples were also taken from each animal. Histologic examination was performed on tissue fixed in 10% buffered formalin, routinely processed and subsequently embedded in paraffin. Sections were stained with hematoxylin and eosin (H&E). Additionally, to assess angiogenesis, immunohistochemical staining using anti-CD31 antibody (Biorbyt Ltd., UK) as a marker for neovascularization on the endothelial surface of skin vasculature as reported previously (6, 23). In each section, a total of 10 different fields in one flap section at 400× magnification were randomly selected, and the vessel number were counted. The vessel density was calculated as number of vessels per square millimeter field (0.55 mm^2^ each field), and the mean was reported for each animal (6, 23).

Double immunohistochemistry for IL-10/VEGF and IL-10/HGF was performed using the BOND-III fully automated IHC and ISH stainer (Leica Biosystems, USA) according to the manufacturer's instructions. In brief, paraffin-embedded tissue sections were first dewaxed and rehydrated, followed by epitope retrieval (epitrope-retrieval solution 2; Leica). They were then incubated with the primary anti-hIL-10 antibody (Thermo Scientific) at 1:100 dilution for 15 min, followed by a post-primary-IgG-linker and a Poly-AP-IgG reagent (Bond Polymer Refine Red Detection System, Leica). Sections were then developed in Fast Red substrate chromogen (Leica). Then the stained sections of IL-10/VEGF and IL-10/HGF groups were incubated with anti-hVEGF antibody (Abcam, USA) and anti-hHGF antibody (R&D Systems, UK) at 1:100 dilution, for 15 min, respectively. This step was followed by a post-primary-IgG-linker and a Poly-AP-IgG reagent (Bond Polymer Refine Red Detection System, Leica, Germany). The second staining of the sections were developed in 3,3-diaminobenzidine (DAB), according the manufacturer's instructions (Leica, Germany).

#### Fluorescence Lifetime Imaging of NADH in *ex vivo* Sections

To evaluate the effect of treatment on cell metabolism, label-free fluorescence lifetime imaging (FLIM) was performed on unstained, unfixed frozen sections (20 μm) of the healing epithelium. Fluorescence lifetime is a measure of the time between fluorescence excitation and emission, and can distinguish between free and mitochondrial-bound NAD (14). Fluorescence lifetime data was acquired with a multi-photon microscope (Bruker Ultima Investigator; Middleton, Wisconsin) equipped with a Becker and Hickl SPC-150 card (Becker and Hickl, Berlin) and a Ti:Sapphire laser (Mai Tai, Spectra-Physics; Santa Clara, California). All images were acquired with a 20×, 1.0 NA water immersion objective (Olympus; Tokyo, Japan) at 512x512 pixel resolution (584 × 584 μm). NADH fluorescence was isolated using a 460 (±20) nm filter (Chroma, ET460/40m-2p) and 755 nm excitation, and a fluorescence lifetime decay histogram was generated at each pixel through time-correlated single photon counting over a 2-min integration time.

#### Phasor Analysis of Bound/Unbound NADH

To analyze the NADH fluorescence lifetime decay curves, a phasor analysis approach was employed as previously described (13, 14). Phasor analysis allows for a simple cluster visualization of fluorophores that contain different molecular species or binding states, such as bound and unbound NADH (13). Using custom-written MATLAB code, fluorescence lifetime decays from each pixel were transformed into respective cosine and sine components to create G and S phasor coordinates plotted within the unit circle designated by *S* = (*G*(1–*G*))^1/2^ (13, 14). Using this transform, each pixel of the FLIM image can be mapped to a phasor coordinate (*G, S*), where (1, 0) corresponds to a lifetime of 0, and (0,0) corresponds to ∞. The instrument response function was measured using second harmonic generation of 1.0 M urea crystals, and deconvolved from the measured fluorescence lifetime decay to improve the accuracy of phasor coordinates at each pixel (24). The epithelium was manually traced from each FLIM imaged, and the average (*G, S*) coordinates from the epithelial pixels in each image were computed. Higher values of *G* correspond to epithelia with shorter NADH lifetimes (more free NADH), while lower values of *G* correspond to longer lifetimes (more protein-bound NADH).

### Statistical Analysis

Analyses were conducted using the GraphPad Prism version 6.01 (GraphPad Software, Inc., USA) and JMP Pro 13 (SAS Institute, USA). Descriptive statistics were presented for the animals in mean ± SD. Mann–Whitney test was used to detect differences between groups. Differences in phasor coordinates, *G* and *S*, were assessed using a one-way ANOVA and *post-hoc* Dunnett's test. The ANOVA design considered individual tissue sections as a random effect nested within each rat. All *p*-values relate to two-sided tests with an alpha level of 0.05.

## Results

### Efficacy of Combined *in vivo* Electroporation-Mediated Gene Transfer on Flap Necrosis

**Figure 1:**
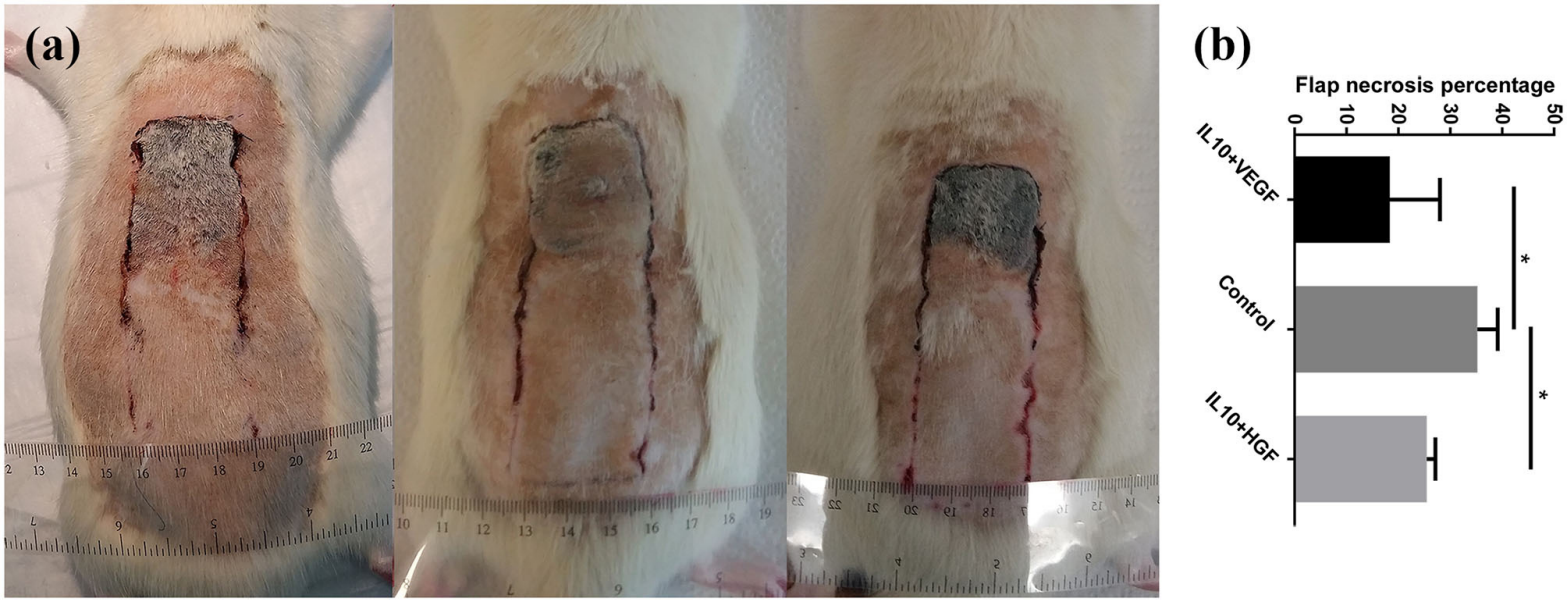
**(a)** Comparison of tissue necrosis among control group, IL-10/ VEGF group, and IL-10/ HGF group. **(b)** Significantly, reduced necrosis was detected in the experimental groups compared to the control group. Data are presented as mean with SEM *p = 0.0079.

Clinical assessment of the animals 7 days after the surgery showed skin ischemic necrosis only in the distal portion of the skin flaps. Combined electroporation mediated IL-10 and HGF gene delivery decreased flap necrosis percentage compared to the control group significantly (Flap necrosis percentage: 25.49 ± 1.65% vs. 35.23 ± 3.90%; *p* = 0.0079, respectively). Furthermore, application of electroporation mediated IL-10 and VEGF gene transfer caused a significant improvement of the flap survival (Flap necrosis percentage: 18.34 ± 9.70 vs. 35.23 ± 3.90%; *p* = 0.0079, respectively; Figure 1).

### Histology Analysis of Skin Flap and Safety Assessment

Caudal sections (more healthy zone) showed a regularly-stratified epithelium with ordinary developed hair follicles. Cranial sections (necrotic zone) were similar in the control and experimental groups and showed an inflammation with monocytes and neutrophils.

**Figure 2:**
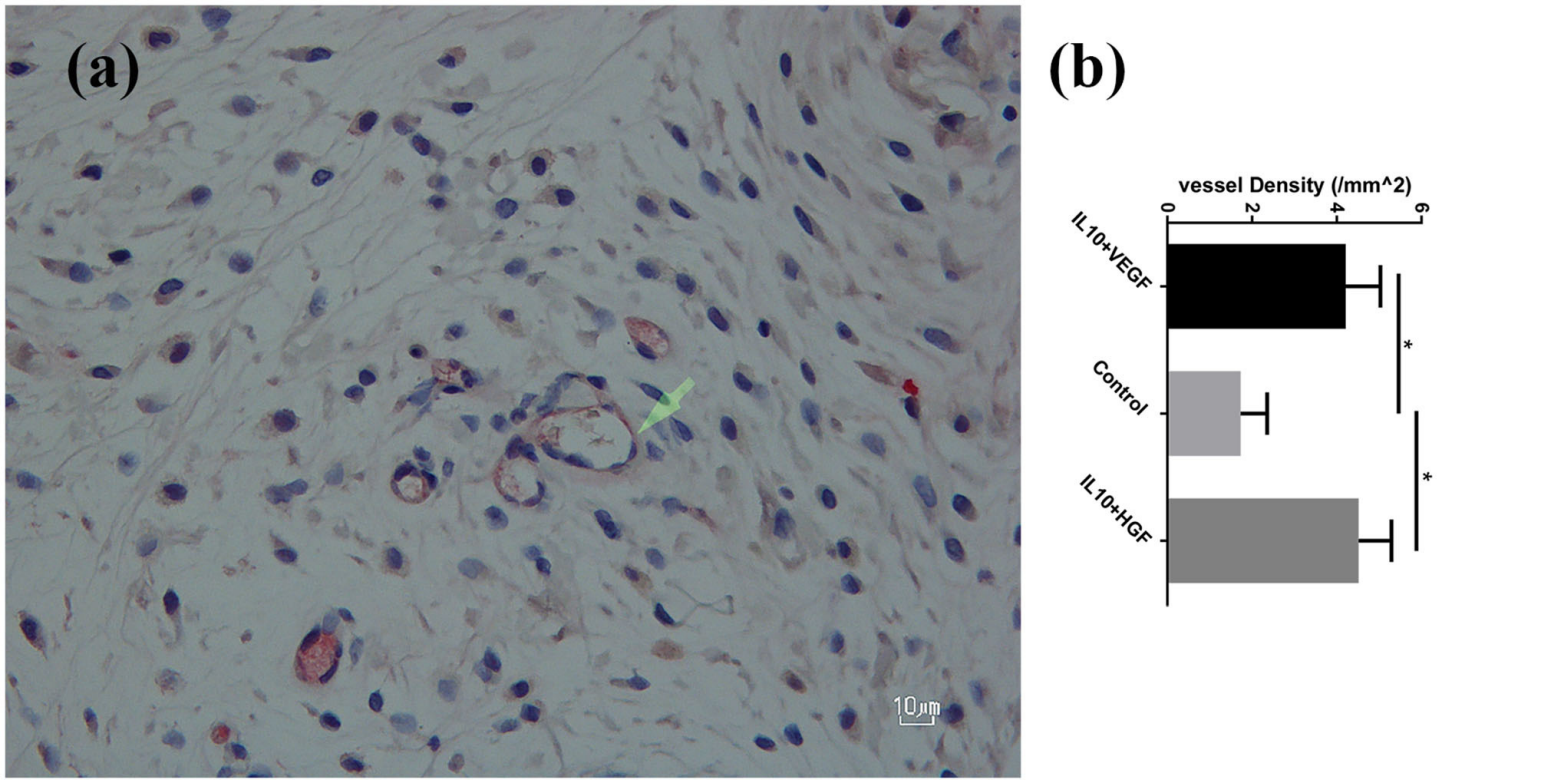
**(a)** CD 31^+^ vessels in the experiment group-streptavidin/alkaline phosphatase staining; 400× magnification. **(b)** Significantly higher vessel density was detected in the experimental groups compared to the control group. Data are presented as mean with SEM *p = 0.0079.

**Figure 3:**
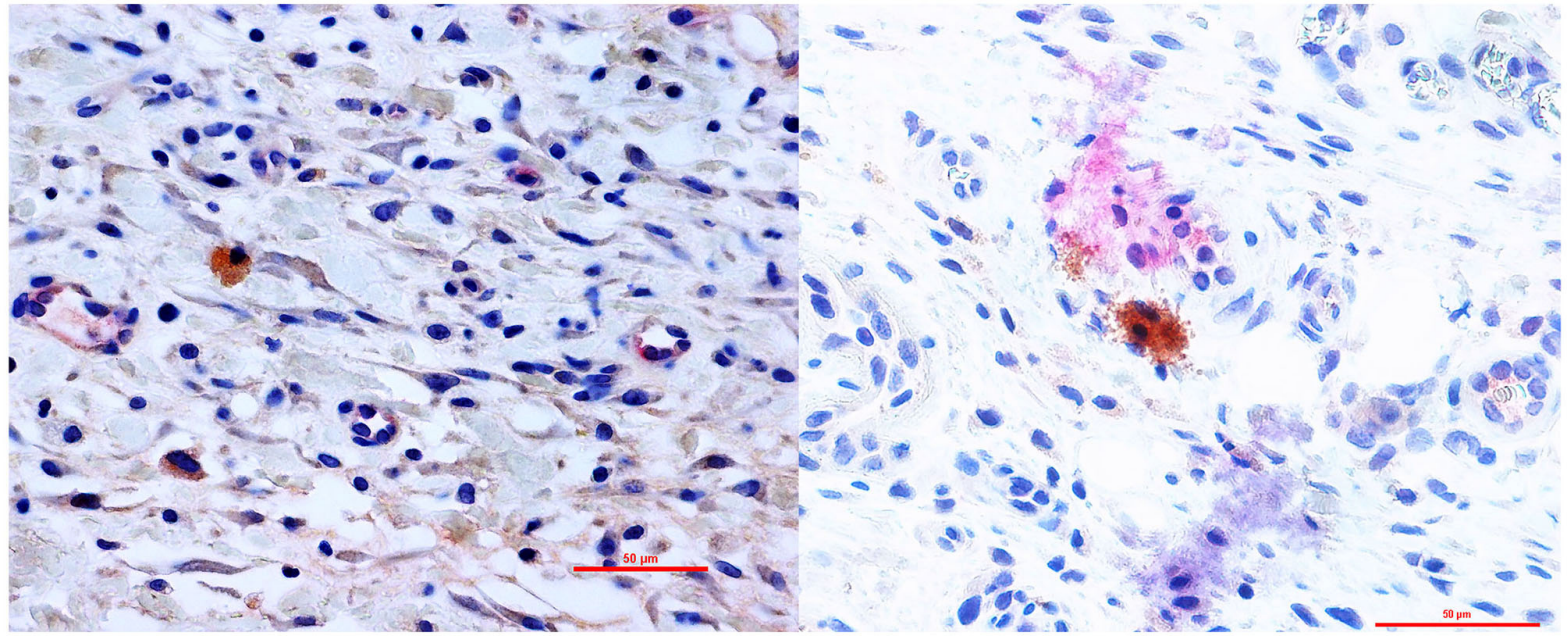
Double immunohistochemistry staining showed protein expression of the delivered genes in the flap. Right: IL-10/ HGF double staining- Primary Ab: anti-hHGF antibody (brown) and anti-IL-10 antibody (Red) 400× magnification. Left: IL-10/ VEGF double staining- Primary Ab: anti-hVEGF antibody (brown) and anti-IL-10 antibody (Red) 400× magnification.

Furthermore, a significantly higher mean vessel density was detected in the group B (IL10/HGF) and group C (IL-10/VEGF) compared group A (control group) (Mean vessel density (/mm^2^): 4.52 ± 0.78 and 4.21 ± 0.82 vs. 1.73 ± 0.62; *p* = 0.0079, respectively; Figure 2). Additionally, double immunohistochemistry staining showed relevant protein levels as the product of the delivered genes (Figure 3).

In the clinical evaluation 7 days after the surgery, no skin damages were observed following electroporation mediated gene delivery. Furthermore, assessment of liver and kidney samples by the veterinary pathologist did not show any significant histologic findings in the therapeutic groups compared to control animals.

### Metabolic Imaging of NADH Fluorescence Lifetime

**Figure 4:**
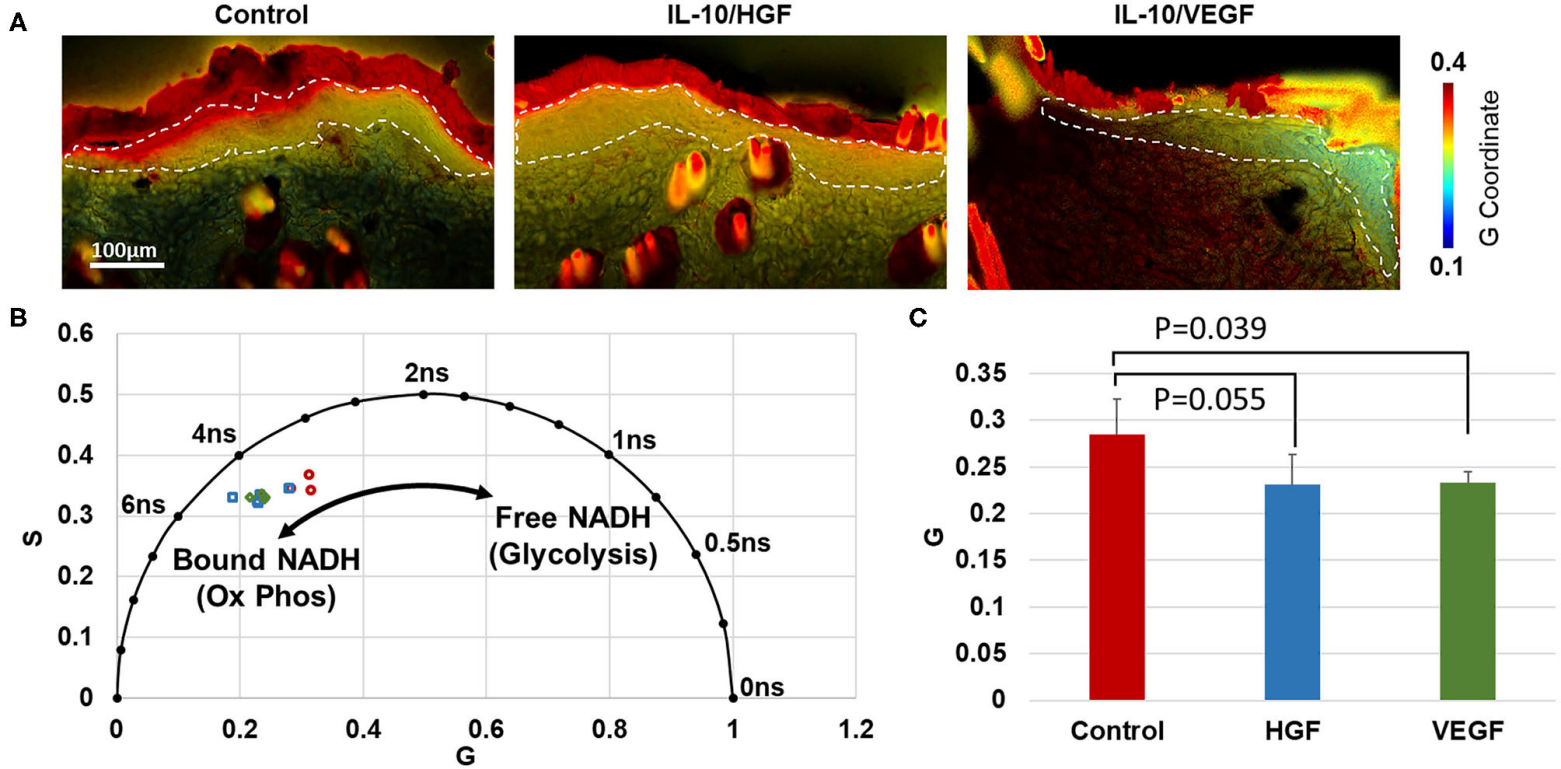
Phasor analysis of NADH fluorescence lifetime in the healthy region of the flap indicates increased oxidative phosphorylation in the epithelium of treated groups. **(A)** Phasor plots demonstrate the NADH lifetime at every pixel in each image. Color regions correspond to the fluorescence lifetime from the epithelium. **(B)** Average phasor coordinates of the epithelium in each rat indicate that treated flaps contain less free NADH, suggesting less glycolysis than control rats. **(C)** A significant difference in the average phasor coordinate (G) was identified between IL-10/VEGF treatment and control samples.

Through phasor analysis of the NADH fluorescence lifetime decay, two molecular lifetime species of NADH were inferred based on the variability in coordinate positions along one axis (Figure 4). Relative to the control group, the epidermis of IL-10/VEGF treated skin had a lower G coordinate (*p* = 0.039). IL-10/HGF treatment had a similar, but not significant, effect on the phasor coordinates (*p* = 0.055). The lower G coordinates in the treated samples indicates a longer lifetime of NADH, which is consistent with a shift toward more mitochondrial-bound NADH in the epidermis. An increase in bound NADH has generally been taken as an increase in oxidative phosphorylation relative to glycolysis (13–15).

## Discussion

In the current study, a combined *in vivo* electroporation-mediated gene transfer for one gene (IL-10) before the surgery and second (VEGF or HGF) after the surgical procedure is shown as a novel approach for management of ischemic skin flap. This synergistic effect resulted in reduction of necrosis, improvement of neovascularization and acceleration of wound healing process. Moreover, no significant adverse findings were recorded in the skin, kidney or liver of the treated animals.

Protective effect of IL-10 gene transfer on survival of skin flaps is due to the fact that overexpression of IL-10 recapitulates fetal-like scar less wound healing capability in postnatal tissue (25). In addition, further beneficial effects of IL-10, such as regulation of the extracellular matrix, attenuation of the inflammatory response, induction of hyaluronan production, improvement of fibroblast function, and increase in endothelial progenitor cells can further justify anti-fibrotic and anti- necrotic effect of IL-10 (25–29). As demonstrated before, application of combined gene transfers of growth factors to wounds can enhance the rate of healing (30). VEGF (Vascular endothelial growth factor) gene therapy (mediated by various viral and non-viral gene delivery methods) has been shown to improve the survival of various skin and muscle flaps (31–33), related to actions like VEGF induced vasodilation, endothelial cell proliferation, neovascularization, apoptosis and increase of glucose transport across the endothelial cell membrane (2, 8, 34–39). Furthermore, HGF (Hepatocyte growth factor) as a potent angiogenic factor can stimulate the production of vessels by promotion of vascular smooth muscle cells migration and endothelial proliferation (40–42). Since wound healing is a complex process, dynamic approach is required to enhance healing and tissue remodeling; therefore combinatorial effect of the gene transfer before and after surgery demonstrated promising results by synergistic and supportive biological mechanisms.

The effects of pro-angiogenic factors may be detectable through NADH FLIM imaging. In an ischemic environment, the lack of sufficient oxygen leads to aerobic glycolysis, producing an increase in free NADH and an overall decrease in NADH fluorescence lifetime (13, 15). Through FLIM imaging of frozen tissue section, an overall increase in NADH lifetime with treatment was observed in the epidermis of the flaps, which is indicative of a shift from glycolytic to oxidative metabolism. These shifts toward oxidative metabolism with treatment may be the result of increased neovascularization. Future work will explore whether *in vivo* NADH FLIM imaging may be able to provide an early marker of flap necrosis and treatment efficacy.

Although no adverse effects related to the therapy were detected among the study animals, we have not evaluated the long-term effect of synergistic gene transfer. Since this is a feasibility study small sample size of the animals were studied and different therapy combinations could not administered and evaluated. Nevertheless, the results of the current pilot study demonstrated the feasibility of the electroporation mediated combined gene transfer as a simple and safe local treatment strategy to improve skin flap survival. However, future studies using a bigger animal model are required to assess the mechanism, efficacy and safety of this method in more details before clinical translation of this promising system.

## Data Availability Statement

The raw data supporting the conclusions of this article will be made available by the authors, without undue reservation.

## Ethics Statement

The animal study was reviewed and approved by Bern cantonal animal experiment commission.

## Author Contributions

SS, FB, HR, AW, MS, TG, KQ, RH, and AG designed the study and performed acquisition, analysis, and interpretation of data. SS, HR, AW, KQ, and AG wrote the manuscript. SS, FB, HR, AW, MS, TG, KQ, RH, and AG performed critical revision of the manuscript for important intellectual content. All authors contributed to the article and approved the submitted version.

## Conflict of Interest

The authors declare that the research was conducted in the absence of any commercial or financial relationships that could be construed as a potential conflict of interest.
